# Intention to voluntary HIV counseling and testing (VCT) among health professionals in Jimma zone, Ethiopia: the theory of planned behavior (TPB) perspective

**DOI:** 10.1186/1471-2458-13-140

**Published:** 2013-02-15

**Authors:** Fira Abamecha, Ameyu Godesso, Eshetu Girma

**Affiliations:** 1 College of Health Sciences, Mizan-Tepi University, Mizan, Ethiopia; 2 Department of Sociology, Jimma University, Jimma, Ethiopia; 3 Department of Health Education and Behavioral Sciences, Jimma University, Jimma, Ethiopia

**Keywords:** HIV/AIDS, VCT, HCT, Health professionals, Intention, TPB, Jimma zone, Ethiopia

## Abstract

**Background:**

Voluntary HIV Counseling and Testing (VCT) forms one of the cornerstones of HIV prevention strategies. It is imperative to understand HIV testing correlates and their theoretical underpinnings in order to promote VCT uptake. The aim of this study was to predict the intention to VCT and associated factors among health professionals in Jimma zone, Ethiopia using the theory of planned behavior.

**Methods:**

An institution based cross-sectional quantitative study among a sample of 336 health professionals in 12 selected districts of Jimma, Ethiopia was conducted in 2012. The constructs and principles of the theory of planned behavior (TPB) were measured. Data were collected using structured questionnaire on self administered basis. A multivariable linear regression model was used to predict the role of independent variables/TPB constructs on the intention to use VCT using SPSS version 16.0.

**Results:**

The components of TPB independently explained the variance in intention to VCT by 30.3%. Both components of TPB and socio-demographic characteristic in the final model explained 32.7% of variance in the intention to use VCT services. Significant proportions (33.0%) of the respondents have never been tested for HIV. The respective indirect components of the TPB predicted the direct components. The strongest predictors of intention to VCT were subjective norm (β=0.39, p<0.001) and attitude (β= 0.19, p<0.001) whereas, none of the socio-demographic variables were significantly predicted the intention to use VCT. Past VCT experience did not have significant statistical association with VCT use intention.

**Conclusions:**

Behavioral intention to use VCT was a function of attitude and perceived social pressure. Demographic related social determinants were not barriers for VCT use intention. Most health workers test their blood by themselves. Strategies to empower health professionals on social pressure resistance and programs targeted at changing negative attitude on VCT use can enhance intention of health professionals to use VCT.

## Background

At the end of 2010, an estimated 34 million people were living with HIV. The number of people dying of AIDS-related causes was 1.8 million and there were 2.7 million new HIV infections. Sub-Saharan Africa remains the region most heavily affected by HIV. About 68% of all people living with HIV resided in sub-Saharan Africa which also accounted for 70% of new HIV infections. The epidemic was most severe in southern Africa, which has more people living with HIV (an estimated 5.6 million) than any other country in the world [1].

With an estimated 1.1 million people living with HIV, Ethiopia has one of the largest populations of HIV-infected people in the world in 2009. In the same year, an adult HIV prevalence was estimated to be 1.8% for males and 2.8% for females [2]. The estimated national incidence rates as inferred from prevalence data to be (1.7%) and (0.46%) for towns and rural areas respectively [3,4].

In Jimma zone; Ethiopia study conducted in 2006 showed that, Out of a total 5648 registered VCT service utilized clients seven 742 (13.1%) were positive for HIV [5] and according to the zone Health department first quarter report, 2011, there were about 1221 peoples living with HIV/AIDS [6]. Furthermore, populations at higher risk of HIV infection were identified to be: sex workers, uniformed services, long-distance-trucker-drivers; refugees and displaced people, daily laborers, mobile/migrant laborers, including cross-border population, street children, high school and university students, out-of-school youth and indigenous populations in remote foreign tourist destinations involved transactional sex [2]. A study in South Africa on occupational hazard in health facilities on 721 health workers, found that 15.7% of them were living with HIV/AIDS [7].

Voluntary HIV counseling and testing has been introduced in many low-resource settings as it helps to create awareness of an individual’s HIV status and offers the opportunity for counseling on risk behavior modification being a cornerstone in the prevention of HIV/AIDS [8-10]. Among the general public in the fight against HIV/AIDS, the behavior of health professionals affects the public’ behaviors and the quality of services provided [10-12]. VCT use by health professionals has generally the following important aspects. First, like any other individuals it is a mechanism for protection of health workers from HIV infection. Second, from the behavior change perspective, health professionals should be role models in undertaking VCT so that it may increase VCT uptake by the general public. Third, due to accidental needle injury and other clinical practices they are vulnerable population for infection. Finally, protecting health workers in developing countries like Ethiopia where health institutions are highly understaffed is much important not to lose highly experienced and qualified health workers.

Hence, in order to promote VCT-uptake, it is imperative to understand HIV testing correlates and their theoretical underpinnings. Theory of planned behavior is a social cognition model (SCM) that constitutes a promising framework for understanding and predicting behaviors and behavioral intentions [13]. In an effort to identify factors associated with the utilization of VCT services, studies have applied theoretical models [14,15] and the other studies have tested the applicability of the theory of planned behavior focusing on sexual behaviors such as intention to use a condom [16,17], intention to refuse sexual debut [18] and intended use of contraception [19,20] in Africa. Thus, theory based studies are used to construct effective and evidence based educational programs that can assist in the control and prevention of the HIV infection [13].

The TPB was applied to health professionals due to the following reasons. First, the HCWs decision making is the result of both intrapersonal and external factors, secondly, it will appears to be logical that the extent to which HCWs participate in the provision and utilization of VCT services is a function of conditions like services, programs, support from other workers. Finally, the normative issues concerning HIV/AIDS are not well established in developing countries like Ethiopia which may leads to stigma and discrimination.

It has been recommended that the TPB is open to the inclusion of other variables if they increase predictive utility of the model [13]. Consistent with this reasoning about the sufficiency of this theory, the current study extended the TPB by adding a measure of perceived risk and perceived severity. Past behavioral experience is also included in to the model because past behaviour often influences the future expressions of that behaviour and thus, increases predictive utility of the model [14-16]. Because of its content and use of repeatedly tested theory, this study is the first of its kind in Ethiopia which may indicate a need for special intervention on health professionals towards VCT. There for the aim of this study was to predict the intention VCT use and associated factors among health professionals in Jimma zone, Ethiopia using the theory of planned behavior.

## Methods

### Study design and settings

Institution based cross-sectional study employing quantitative study triangulated with qualitative study was conducted in Jimma zone, Ethiopia from February 05 to March 28, 2012. Jimma is located in Oromia regional state and 357 kms South West of Addis Ababa. The zone is subdivided in to 18 districts with the total projected population of 2,770,329 from 2007 central statistical agency (CSA) census report.

### Study participants

Health professionals included were at least diploma holders. Private health institutions were excluded to avoid double counting since government health workers may be involved in the private institutions and their number and services are minimal. Participants who were ill and unable to respond were excluded.

### Sampling

Out of 18 districts in the zone, 12 districts were included in this study using simple random sampling technique. Then, all 369 health professionals in the 12 selected districts were included in the study*.* Health professionals working (employee) in government owned health settings (both health centers and health offices) in all selected districts of the zone were included.

### Data collection

Quantitative data was collected using structured questionnaire adapted and modified from previous studies [13,21]. Modification was also done based on results from elicitation study and was translated to local languages, Afan Oromo and Amharic, and back translated to English by language experts. Data were collected using questionnaires of two languages, on self administered basis. In addition to the cognitive variables, the questionnaire covered socio-demographic information, information on history HIV counseling and testing. Training was provided for data collectors. Elicitation study was conducted on a sample of 15 participants selected from a health center in the study area. Important beliefs for the study population regarding the behavioral consequences of using VCT services, the significant referent and control beliefs for this behaviour were identified through interviews with participants from the target group. They were required to indicate their beliefs regarding advantages and disadvantages following use of VCT services, the people/groups that would approve/disapprove their use of VCT services and factors that would either hinder or facilitate their use of VCT services. Furthermore, pretesting on 5% of sample drawn from unselected districts was conducted prior to data collection to assess the cultural sensitivity and clarity of the items in questionnaires.

### Measurements

Intention to use VCT was measured by using four items. Responses ranged from ‘not likely at all’ (1) to ‘very likely’ (5) (Example: How likely you are to be in need of HIV counseling and testing services the next 3 months?). Direct attitude towards the use of VCT was measured using 4 items on bipolar differential scales. For the belief about the use of VCT, respondents were asked to rate the extent to which they agree to thoughts of using VCT services in the next three months. Six items were used with responses ranged from ‘strongly disagree (1) to ‘strongly agree’ (5). Evaluation of VCT belief was measured by asking of respondents to evaluate six salient consequences accruing from using VCT services. Each behavioral belief was multiplied by the score for the outcome evaluation to create a new variable that represents the weighted score for each behavioral belief. Similarly, we weighted each normative belief by the score for motivation to comply and each control belief by the score representing the influence of the control belief. Then we sum-up the weighted beliefs to create a composite score for indirect attitude, SN and PBC.

Four Likert scale items were used to measure direct subjective VCT norm. VCT intention included intention to pretest counseling, HIV testing, receiving the result of the test and post test counseling. To assess direct NB towards VCT, participants were asked six Likert scale items to indicate the extent to which they thought their spouse/partner, friends, colleague, employer, neighbors, religious leaders and relatives of their pupils were likely to appreciate their use of HIV counseling and testing services. Direct measure of PBC was measured by using 4 items on bipolar differential scales. High composite score shows strong perceived ability or less difficulty to have VCT services within the specified period of time. Perceived risk of HIV and Perceived severity of HIV and respectively were measured using 5 items for each. One item was used to ask respondents whether they have ever been tested for HIV by using yes/No approach.

### Data analysis

Data were checked for completeness, edited, coded and carefully entered by principal investigator for analysis using SPSS-version 16.0. Descriptive Statistical measures like mean and standard deviation were done. Independent sample t-test and correlation analysis between direct and indirect measures of the same constructs (indirect attitude Vs direct attitude, indirect SN Vs direct SN and indirect PBC and direct PBC) was done to confirm the validity of the indirect measures. Using a multiple linear regression procedure, we entered intention as the dependent variable, and the direct measures of attitude, SN and PBC as the predictor variables.

Ethical clearance was obtained from Jimma University College of public health and medical sciences research ethics committee. Letter of support was also obtained from the zonal health department to all selected district health offices. Written informed consent was secured from each participant.

## Results

### Socio-demographic characteristics

A total of 336 health care professionals from 12 districts were participated in the study. The response rate was 91%. Two hundred and fourteen (63.7%) of the respondents were male. The mean age was 27.5 (SD=2.7) years. Concerning marital status, 52.8% were married and 46.0% were single. Majority of respondents were Muslims (36.3%) followed by Orthodox (32.0%). The highest proportion of ethnic group was Oromo 236 (69.4%). Large proportions 113(40.2%) of participants were nurses (Table 1).

**Table 1:** Socio-demographic characteristics of health professionals in Jimma zone, Ethiopia, 2012

**Socio demographic variables**	**Frequency**	**Percent**
**Age category (n=336)**		
21-30	275	81.8
31-40	50	14.9
41-50	11	3.3
**Gender (n=335)**		
Male	214	63.7
Female	121	36.3
**Religion (n=331)**		
Muslim	120	36.3
Orthodox	106	32.0
Protest	89	26.9
Others	16	4.8
**Ethnicity (n=336)**		
Oromo	236	69.4
Amhara	43	12.6
Others (Tigre, Guraghae, Kafa, Wolyita, Dawro and Yem)	57	18.0
**Marital status (n=324)**		
Single	149	46.0
Married	171	52.8
Divorced/separated	4	1.2
**Education (n=329)**		
Diploma	158	48.0
Degree	168	51.1
Masters	3	0.9
**Profession (n=281)**		
Nursing	113	40.2
Health officer	45	16.0
Laboratory	35	12.5
Pharmacy	40	14.2
Midwifery	28	10.0
Environmental health	17	6.0
Others (MPH, DDM)	3	1.1

### Voluntary HIV counseling and testing status

A total of 121 (36.0%) individuals were involved in HIV testing by VCT counselor and received post test counseling. One hundred and six (31.0%) of the participants have tested their HIV status by themselves and 109 (33.0%) of the respondents have never been tested. The most frequently mentioned reasons why respondents received their test results (post test counseling) were ‘because of fear that one could be infected’ 65 (19.0%) and ‘fear of being at risk due to their profession’ 52 (15.3%). Nurses were the highest (31.0%) and Environmental Health professionals were the lowest (1.4%) in proportion to use VCT services. Among the total respondents who got tested for HIV, 86 (37.9%) have tested two times and 74 (32.6%) participants have tested three times and above (Table 2).

**Table 2:** Descriptive statistics for the components of the theory of planned behaviour, perceived risk, perceived severity and intention for health professionals in Jimma zone, Ethiopia, 2012

**Components**	**N**	**Items**	**Scale range**	**Scale mean (SD)**
Direct SN	336	4	4-20	14.45 (3.37)
Intention	336	4	4-20	14.55 (3.82)
Direct PBC	336	4	4-28	19.40 (6.14)
Direct Attitude	336	4	4-28	21.26 (5.19)
Perceived severity	335	5	5-25	15.37 (5.06)
Perceived risk	335	5	5-25	15.75 (4.76)
Behavioral belief (BB)	336	6	6-30	24.47 (4.24)
Evaluation of behavioral belief (EBB)	333	6	6-30	21.26 (5.36)
*Indirect attitude=∑(BB)*_*i*_*(EBB)*_*i*_	336	6	6-150	105.63 (30.17)
Normative belief (NB)	336	6	6-30	21.82 (5.21)
motivation to comply (MC)	336	6	6-30	19.15 (5.74)
*Indirect SN=∑(NB)*_*i*_*(MC)*_*i*_	336	6	6-150	80.99 (32.99)
Control belief (CB)	335	6	6-30	22.85 (5.19)
power of control (PC)	336	6	6-30	18.94 (5.87)
*Indirect PBC=∑(CB)*_*i*_*(PC)*_*i*_	336	6	6-150	62.36 (31.72)

Do not add to 336, due to missing cases, *SD*= standard deviation, and *PBC*=perceived behavioral control.

### Indirect attitude, subjective norm and Perceived behavioral control

Indirect attitude had high 105.63 (SD=30.17) mean score. The indirect subjective score was moderate 80.99(SD=32.99). The mean score of the indirect perceived behavioral control measure indicated lower value 62.36(SD=31.72) (Table 2). There was positive correlation between the respective indirect and direct components of the theory of planned behavior (Table 3). Indirect attitude was a predictor variable for the direct attitude (β= 0.44, p<0.001). Indirect subjective norm also predicted direct subjective norm (β= 0.45, p<0.001). There was significant statistical association between indirect and direct PBC (β= 0.25, p<0.001).

**Table 3:** Partial correlations (Pearson's r) among the indirect and direct measures of TPB, Jimma zone, Ethiopia, 2012

**Components**	**DATT**	**DSN**	**DPBC**	**IATT**	**ISN**	**IPBC**
DATT	1					
DSN	0.34^++^	1				
DPBC	0.46^++^	0.39^++^	1			
IATT	0.57^++^	0.39^++^	0.38^++^	1		
ISN	0.24^++^	0.52^++^	0.25^++^	0.46^++^	1	
IPBC	0.18^++^	0.25^++^	0.45++	0.31^++^	0.30^++^	1

Correlation is significant at ++p<0.001, +p<0.05.

### Direct TPB components, perceived threats and intention to VCT use descriptive scores

Direct attitude, subjective norm and PBC had mean scores of 21.26 (SD=5.19), 14.45 (SD=3.37) and 19.40 (SD=6.14) respectively. There was higher intention score 14.55 (SD=3.82) to VCT use among the health professionals. There was also higher mean Perceived risk 15.75 (SD=4.76) and severity 15.37 (SD=5.06) scores of HIV infection among the health professionals (Table 2).

### Multivariate analysis

Hierarchal multiple linear regression analysis was done to see the effects of independent variables mentioned below on the behavioral intention to use VCT. All components of TPB, perceived risk, perceived severity, past behavior experience, socio demographic variables and intention were included. The analysis was performed after controlling for the effects of socio demographic variables and past behavior experience. Accordingly, the socio demographic variables and past VCT experience were entered using the “enter Method” in the first block and explained 6.4% (R^2^ =0.064) of variability in the intention to use VCT. Then all of the components TBP, Perceived risk and Perceived severity were entered in the second block showing the changes in intention by 29.9% (R^2^ =0.299). Some of the socio-demographic factors were significantly associated with intention to VCT in the bivariate analysis but lost their statistical significance in the multiple regression analysis. The PBC and past behavior experience were significantly correlated to intention on a bivariate analysis, but this was no longer sustained in multiple linear regressions analysis.

The statistically significant predictors of VCT use intention in the final model were attitude (β= 0.185, P<0.01) and subjective norm (β= 0.380, P<0.01) (Table 4). This means, for a unit positive change in the individual’s perception about key persons thought them to use VCT service as normative action, will changes the intention to use VCT by 0.38 provided that the other conditions unvaried. At the same time, a unit positive change in the attitude towards the advantage associated with the use of VCT service, will changes the intention to use VCT services by 18.5% keeping the other factors constant.

**Table 4:** Predictors of intention to VCT use on multivariable linear regression analysis of components of TPB, perceived risk and perceived severity among health professionals in Jimma zone, Ethiopia, 2012

**Variables**	**Standardized β coefficients**	**P-values**	**95% confidence intervals**
Constant	3.37	<0.001	[1.96, 5.51]
Direct attitude	0.18	<0.01	[0.05, 0.22]
Direct subjective norm	0.38	<0.001	[0.32, 0.56]
DPBC	0.07	0.22	[−0.03, 0.11]
Perceived risk	0.03	0.55	[−0.06, 0.12]
Perceived severity	−0.03	0.58	[−0.11, 0.06]

*DPBC = Direct perceived behavior control.

Components of the TPB plus perceived risk and perceived severity explained the variability in intention to VCT by 29.9% (R^2^=0.299). Here, the addition of external constructs did not increase the predictive power of the TPB, because the components of the theory independently explained 30.3% of variance in intention to VCT which is greater than 29.9%.

## Discussion

The present study showed that have never been tested and there is high intention to use VCT services. Most health workers test their blood by themselves which may indicate the existence of fear of disclosure of HIV status and high perceived risk of HIV infection.

Like that of studies in Ethiopian and Tanzanian [6,12,22], this finding revealed no significant statistical association between socio-demographic factors and intention to use VCT. This finding has an implication that VCT services are accessible, available and affordable to the health professionals that the social determinants of health are not barriers for VCT use intention. The socio-demographic factors are not predictors since as part of primary health care, VCT services are provided in every health centers in the study area for free for everyone. VCT campaigns are also common that may increase accessibility of VCT services.

Findings of the present study suggested no statistical difference among the age group in requesting for VCT which contradicts with studies conducted in Tanzania and Ghana showing significant differences among age group with likely to be tested among teenagers due that high HIV prevalence among them and increasing tendency for young people to be tested before marriage [15,17]. This difference might be due to that, most of the respondents in this study were married and the low Perceived risk to HIV in this age category might have hampered the association.

In line with the previous Nigerian study on medical students [23] the present study depicted insignificant difference among the two sexes in willingness to seek VCT services. But the current finding was not similar with other studies [24,25]. It is conventional that the gender differences in seeking VCT are mainly due to factors like cultural, social and biological like male dominance, vulnerability to risk, social factors like rape especially in developing countries, that make female vulnerable to HIV infection.

In this study, the level of education might have influenced the socio-cultural factors positively that may leads to the differences among male and female, hence reducing the differences in seeking VCT services. Therefore, the insignificant gender differences in this aspect may be due to the social status of female HCWs is different from that of females in the general population.

The intention to use VCT services was primarily due to subjective norms and attitude while their perceived behavioral control was statistically insignificant predictor. Other studies also found similar results that subjective norms were more important predictors [24,25]. However, other studies showed that perceived behavior control was the leading predictor of intention followed by attitude and subjective norm [23,26]. In present study subjective norm was more important predictor than the other components of TPB which indicated that significant others have great role to play in the use VCT services. The possible reason for this variation could be due to variation across behavior, population and situation under which the behavior is occurring, according to the TPB perspective [13,22]. Given the population for this study were HCWs they could have high perceived behavioral control on VCT. Unlike the general public, the HCWs are expected to have also the knowledge and skill related to HIV/AIDS including VCT.

In assessing the effect of past history of VCT use on the behavioral intention, the present study did not find a significant association after controlling for the effects of socio- demographic variables and components of TPB. This is contrary to previous studies [20,27] and agrees with the assumption of the TPB [13,22] which says past behavior experience affects the future expression of the behavior provided that it must be mediated by the proximal TPB components. The possible reason may be due to that the many HCWs have tested themselves for HIV which may not reflect the actual intention to VCT use.

In this study, none of the external to TPB variables significantly predicted intention to VCT. The finding of this study will goes with a study in Zimbabwean [28] in which the perceived risk was not associated with behavioral intention. The possible explanation related to this may be that HCWs are expected to have high knowledge about HIV/AIDS, unlike the other population group leads to the insignificant association to intention because motivation for VCT might be driven by knowledge and education of VCT itself rather than risk perception for this group. However, previous studies [29-31] claimed that high risk group tends to be less likely to participate in VCT services.

As suggested by the principle of the TPB [13,22], there was positive correlation between the respective indirect and direct components of the theory of planned behavior. Hence, the indirect measures predicted the respective direct measures of attitude, subjective norm and perceived behavioral control. This implies that interventions can be designed on the salient beliefs identified during the elicitation study; so that, by influencing the direct measures of the TPB, intention to VCT use can be increased. The components of TPB explained intention to use VCT in a similar manner with studies of other public health topics in Ethiopia and other African countries [16-18,29]. Since this study was conducted among the health care workers, there may be social desirability bias that could have affected not only the main study but also the elicitation study.

## Conclusions

The study revealed that behavioral intention to use VCT was a function of attitude and perceived social pressure (SN). Demographic related social determinants were not barriers for VCT use intention. Most health workers test their blood by themselves which may indicate the existence of fear of disclosure of HIV status and high perceived risk of HIV infection. The belief based components (indirect measures) of the TPB can influence the direct measures of the TPB so that intention to use VCT will be increased. Strategies to empower health professionals on social pressure resistance and programs targeted at changing negative attitude on VCT use can enhance intention of health professionals to use VCT.

## Competing interests

The authors declare that they have no competing interests.

## Authors’ contributions

FA, AG and EG designed the study, analyzed the data, drafted the manuscript and critically reviewed the article. All authors read and approved the final manuscript.

## Pre-publication history

The pre-publication history for this paper can be accessed here:

http://www.biomedcentral.com/1471-2458/13/140/prepub
